# Palmar Neurofibromas Radiologically Mimicking Lipomas: A Case Series of Surgical Pitfalls and the Role of Immunohistochemistry

**DOI:** 10.7759/cureus.114104

**Published:** 2026-08-07

**Authors:** Hemant Saraiya

**Affiliations:** 1 Department of Plastic Surgery, Gujarat Cancer and Research Institute (GCRI), Ahmedabad, IND; 2 Department of Plastic and Reconstructive Surgery, Saraiya Plastic Surgery Hospital, Ahmedabad, IND

**Keywords:** immunohistochemistry, lipoma, mri, nerve-sparing surgery, palmar neurofibroma, peripheral nerve sheath tumor, ultrasonography

## Abstract

Palmar neurofibromas are rare peripheral nerve sheath tumors (PNSTs) that can radiologically mimic lipomas, posing a risk of unanticipated nerve injury during excision and creating diagnostic and surgical challenges. A retrospective analysis of prospectively maintained data from three consecutive patients with histopathologically confirmed palmar neurofibromas treated between 2017 and 2025 was performed after institutional ethical approval. Two female and one male patient, aged 28-68 years, presented with progressively enlarging and painful palmar swellings. Preoperative ultrasonography and/or magnetic resonance imaging suggested lipomatous lesions in all cases. All patients underwent magnification-assisted nerve-sparing excision, and histopathological evaluation supplemented with immunohistochemical markers, including S-100, SOX10, CD34, and Ki-67, established the diagnosis of neurofibroma in each case. Intraoperatively, a neural origin was identified in all lesions. Meticulous microsurgical interfascicular dissection was carried out to preserve nerve continuity. Despite misleading radiological appearance, complete excision with preservation of the involved nerves was achieved in all patients. During follow-up ranging from one to eight years, no recurrences were observed; one patient experienced transient postoperative digital numbness that resolved completely within three months. Palmar neurofibromas are uncommon lesions that may be difficult to distinguish from lipomas on imaging, and definitive diagnosis relies on histopathological examination supported by targeted immunohistochemistry to differentiate them from schwannoma, lipoma, and malignant PNSTs. Heightened preoperative suspicion, appropriate patient counseling, meticulous microsurgical technique, and nerve-sparing surgery are essential for optimizing functional outcomes. Surgeons should maintain a high index of suspicion for neural tumors when evaluating palmar soft-tissue masses with apparently benign lipomatous imaging characteristics.

## Introduction

Soft tissue tumors of the hand are uncommon and encompass a wide spectrum of benign and malignant lesions arising from adipose, fibrous, vascular, neural, or muscular tissues. Lipomas are the most common adipocytic tumors of the hand, accounting for approximately 1%-3.8% of hand tumors [1]. Peripheral nerve sheath tumors (PNSTs), including schwannomas and neurofibromas, comprise approximately 5%-15% of upper extremity soft-tissue tumors [2]. Isolated palmar neurofibromas are exceptionally uncommon, with the literature largely limited to isolated case reports and small case series [3-5]. Most are diagnosed only after histopathological evaluation.

Clinical examination supplemented by ultrasonography or magnetic resonance imaging (MRI) constitutes the standard diagnostic pathway for palmar soft-tissue lesions. However, overlapping imaging features [6], particularly between lipomas and PNSTs, limit the diagnostic accuracy. Because a lesion presumed to be a simple lipoma may, in fact, arise from a nerve, an unrecognized neural origin exposes the patient to the risk of iatrogenic fascicular injury and sensory or motor deficits during otherwise routine excision. Definitive diagnosis ultimately relies on histopathological evaluation, with immunohistochemistry (IHC) [7] serving as a vital adjunct for tumor classification and exclusion of malignant mimics.

This study describes three cases of palmar neurofibromas initially interpreted radiologically as lipomas and analyzes the associated diagnostic pitfalls, surgical considerations, histopathological features, immunohistochemical profile, and long-term functional outcomes following nerve-sparing excision. To our knowledge and based on a focused literature search, no prior report has specifically examined the radiologic mimicry of palmar neurofibromas as lipomas while simultaneously analyzing immunohistochemical characteristics and functional outcomes following nerve-sparing excision. This surgical case series was prepared in accordance with the Preferred Reporting Of CasE Series in Surgery 2020 guideline [8].

## Case presentation

All procedures were performed under regional or general anesthesia with an arm tourniquet inflated to 250 mmHg after limb exsanguination, through a longitudinal incision directly over the tumor. Exposure was performed under ×4 loupe magnification. Once the tumor origin from the nerve was confirmed, the parent nerve was traced proximally and distally to identify the entering and exiting fascicles. Careful interfascicular dissection was performed using microsurgical instruments to preserve functional fascicles. In each case, the tumor was intimately associated with a named nerve branch but was separable from the main functional fascicles; no functional fascicle was deliberately sacrificed, and only tiny nonfunctional twigs entering the tumor were divided where unavoidable. Complete excision was defined as the macroscopic removal of the entire lesion with an intact capsule while maintaining continuity of the parent nerve. None of the patients required nerve resection or reconstruction. The excised specimens were processed for histopathology and IHC.

Case 1

**Figure 1:**
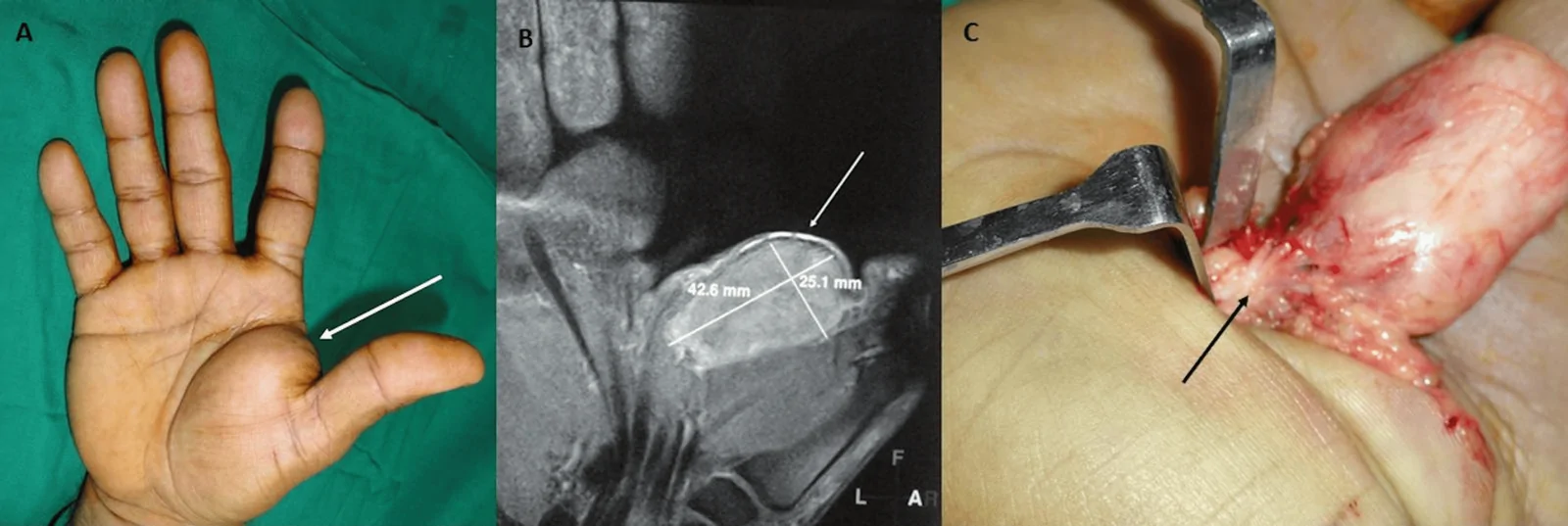
Clinical, radiological, and intraoperative findings of a right thenar swelling (A) Swelling in the thenar region extending into the first web space. (B) MRI demonstrating a well-capsulated, lobulated lesion measuring 42.6 × 25.1 mm with incomplete fat suppression. (C) Intraoperative view showing origin of tumor from a muscular branch of the median nerve MRI: magnetic resonance imaging

A 30-year-old right-handed man presented with a progressively enlarging swelling over the thenar aspect of the right palm for six months. There was no history of comorbidities such as diabetes mellitus, hypertension, or prior trauma. The swelling was initially painless but had recently become associated with localized discomfort and deformity. Clinical examination revealed a firm, well-circumscribed, nontender mass on the palm. It measured approximately 4 × 2.5 cm, with intact overlying skin (Figure 1A). The thumb functions were normal.

MRI of the right hand revealed a well-capsulated, lobulated lesion in the thenar compartment. On axial and coronal images, the lesion was isointense to mildly hyperintense relative to the muscle on T1-weighted sequences and heterogeneously hyperintense on T2-weighted/proton density fat-saturated sequences. Notably, the lesion did not demonstrate complete signal suppression on fat‑saturated sequences, a pattern atypical for a simple lipoma. Following contrast administration, mild heterogeneous enhancement was observed. These features, in retrospect, were more consistent with a PNST (Figure 1B). However, intraoperatively, the lesion was found to be arising from a muscular branch of the median nerve; the nerve was seen entering and exiting a fusiform mass, with fascicles splayed over the capsule, confirming the neural origin. Magnification-assisted microsurgical dissection enabled complete excision while preserving the continuity of the involved nerve (Figure 1C).

Histopathological examination revealed a spindle cell lesion with wavy nuclei embedded in a collagenous stroma. IHC examination revealed diffuse S100 and nuclear SOX10 positivity. CD34 was not performed for this case. Histopathology and IHC confirmed the diagnosis of neurofibroma.

**Figure 2:**
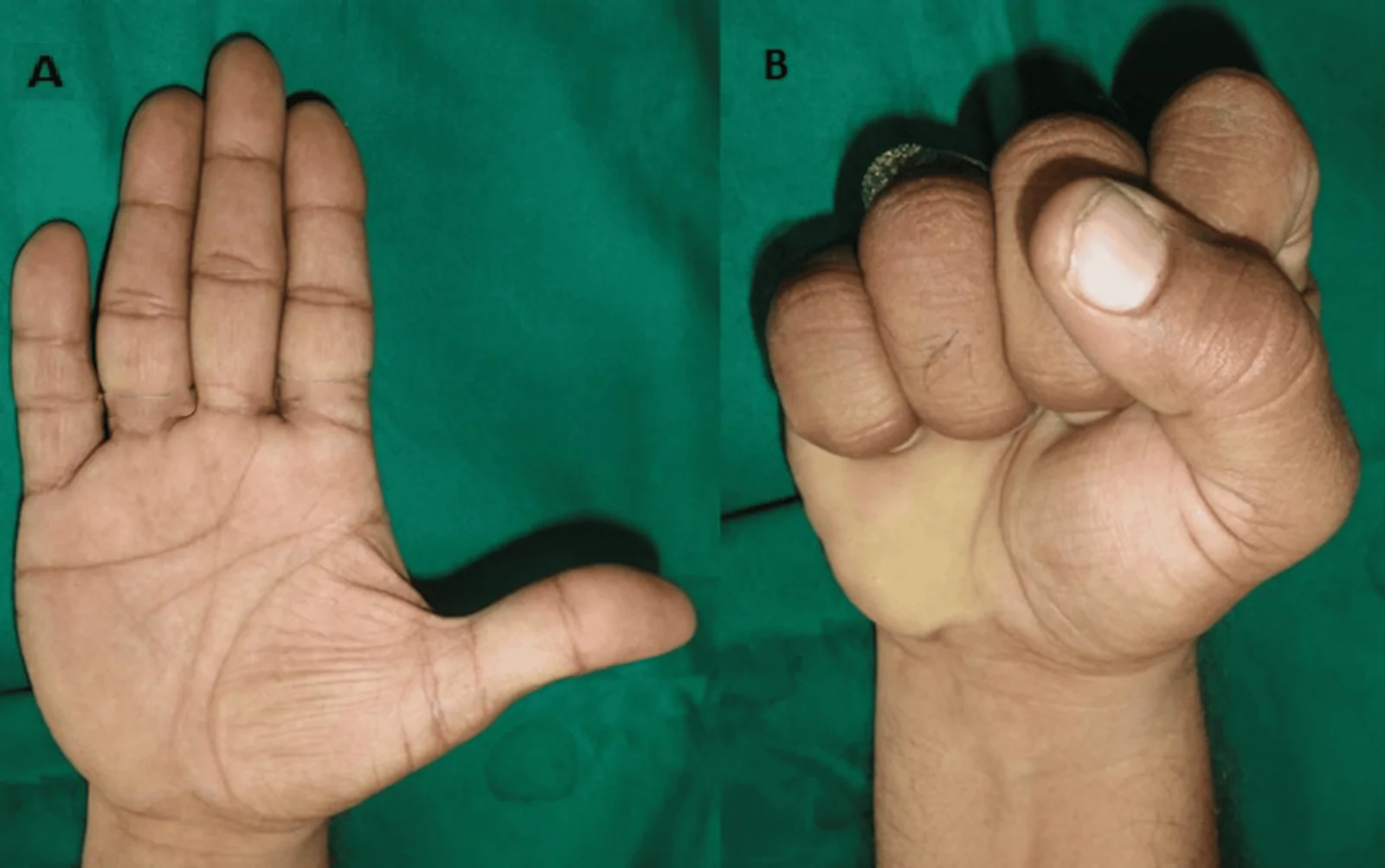
Postoperative outcome of the thenar swelling (A) No evidence of recurrence at follow‑up. (B) Full restoration of hand function with no neurological deficit

Histopathological images, antibody clone, dilution, and automated-platform details were unavailable for this case because the original slides and specimen were disposed of in accordance with the pathology department’s routine archival and disposal policy. The patient achieved full hand function without sensory or motor deficits (Figures 2A, 2B). During the eight years of follow-up, there was no evidence of recurrence or neurological impairment.

Case 2

**Figure 3:**
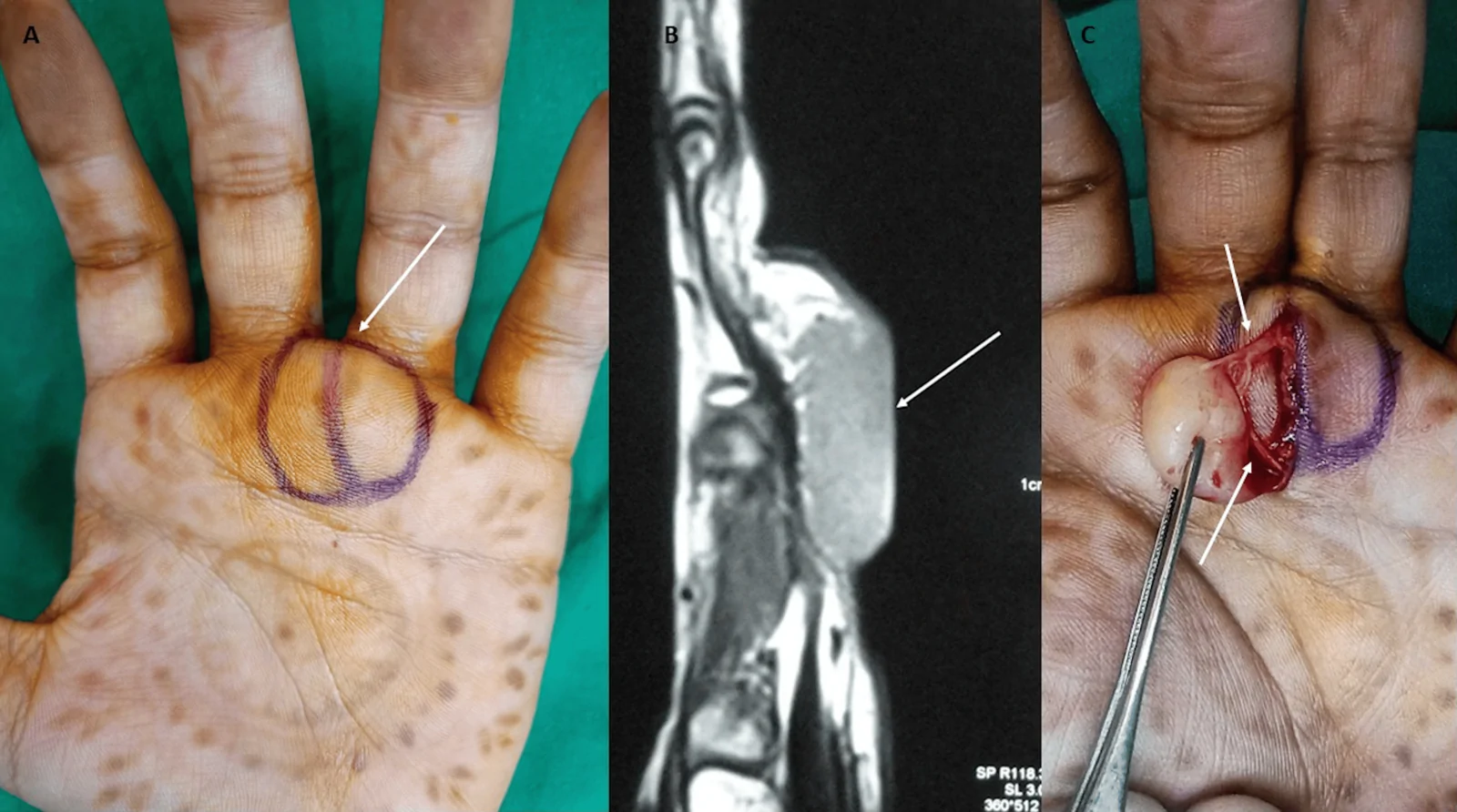
Clinical, radiological, and intraoperative findings of the palmar swelling at the base of the ring finger (A) Swelling at the base of the ring finger encroaching into the web space. (B) MRI showed a well-encapsulated iso-to-hypointense (T1) lesion with incomplete fat suppression in the palmar region at the base of the ring finger. (C) Intraoperative view showing the origin of a tumor from a branch of the digital nerve MRI: magnetic resonance imaging

A 28‑year‑old woman presented with a swelling in the distal palm, located near the base of the ring finger (Figure 3A). The lesion had been first noticed eight months earlier and had gradually increased in size, becoming painful over time. On examination, the mass measured approximately 3 × 3.5 cm, was firm in consistency, was well‑defined, and elicited tenderness on deep palpation. The patient had no significant comorbidities, medication use, or family history of neurocutaneous disorders. Distal neurovascular function was normal.

MRI revealed a well-defined, iso- to hypointense, well-encapsulated lesion in the palmar region at the base of the ring finger. On T1-weighted images, the lesion was iso- to hypointense to muscle and iso- to mildly hyperintense on T2-weighted/fat-saturated images, without uniform T1 hyperintensity or complete fat suppression (Figure 3B). Intraoperatively, however, the lesion was found to be of neural origin and was in continuity with the nerve, separable from the functional fascicles (Figure 3C). Magnification-assisted microsurgical dissection enabled complete excision while preserving the continuity of the involved nerve.

**Figure 4:**
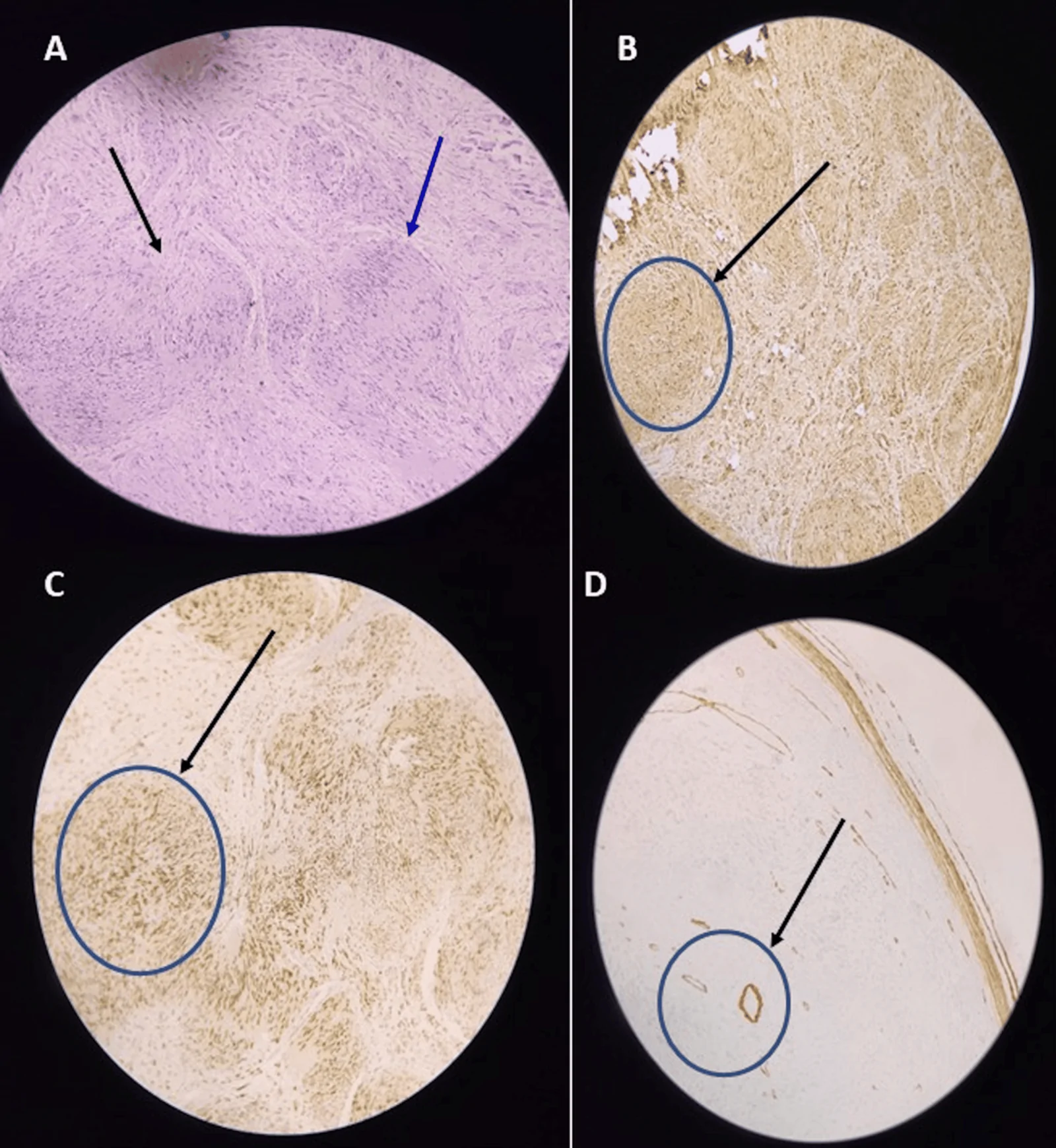
Histopathological picture and images of IHC staining (A) The blue arrow shows spindle-cell proliferation with elongated, wavy nuclei in a collagenous stroma, containing scattered Wagner-Meissner-like (tactile) bodies, while the black arrow shows nerve fibers after staining the excised tissue from the palm with hematoxylin and eosin stain and viewing with 10× magnification. (B) The circle with an arrow shows neurofibroma cells with S100 IHC stain. (C) The circle with an arrow shows nuclear positivity with SOX10 IHC stain. (D) The circle with an arrow shows the SMA IHC stain, which is not present in tumor cells but is present in the blood vessel wall SOX10 and S100 are immunohistochemical markers IHC: immunohistochemistry; SMA: smooth muscle actin

Histopathological examination revealed a spindle-cell lesion with elongated, wavy nuclei in a collagenous stroma containing scattered Wagner-Meissner-like bodies and residual nerve fibers (Figure 4A). IHC showed nuclear SOX10 and S100 positivity in tumor cells, confirming the diagnosis of neurofibroma (Figures 4B, 4C), while smooth muscle actin (SMA) was negative in tumor cells but positive in blood vessel walls as an internal control (Figure 4D), thereby excluding a myogenic or perivascular lesion; CD34 was not performed. The tests were conducted using the Dako Autostainer Link 48 (version 4.2.5; Agilent Technologies, Santa Clara, CA): SOX10 (EP268 clone, schwannoma control) demonstrated nuclear positivity in bundled tumor cells, S100 (Beta EP32 clone, schwannoma control) exhibited both cytoplasmic and nuclear staining, and SMA (1A4 clone, leiomyoma control) confirmed negativity in tumor cells.

**Figure 5:**
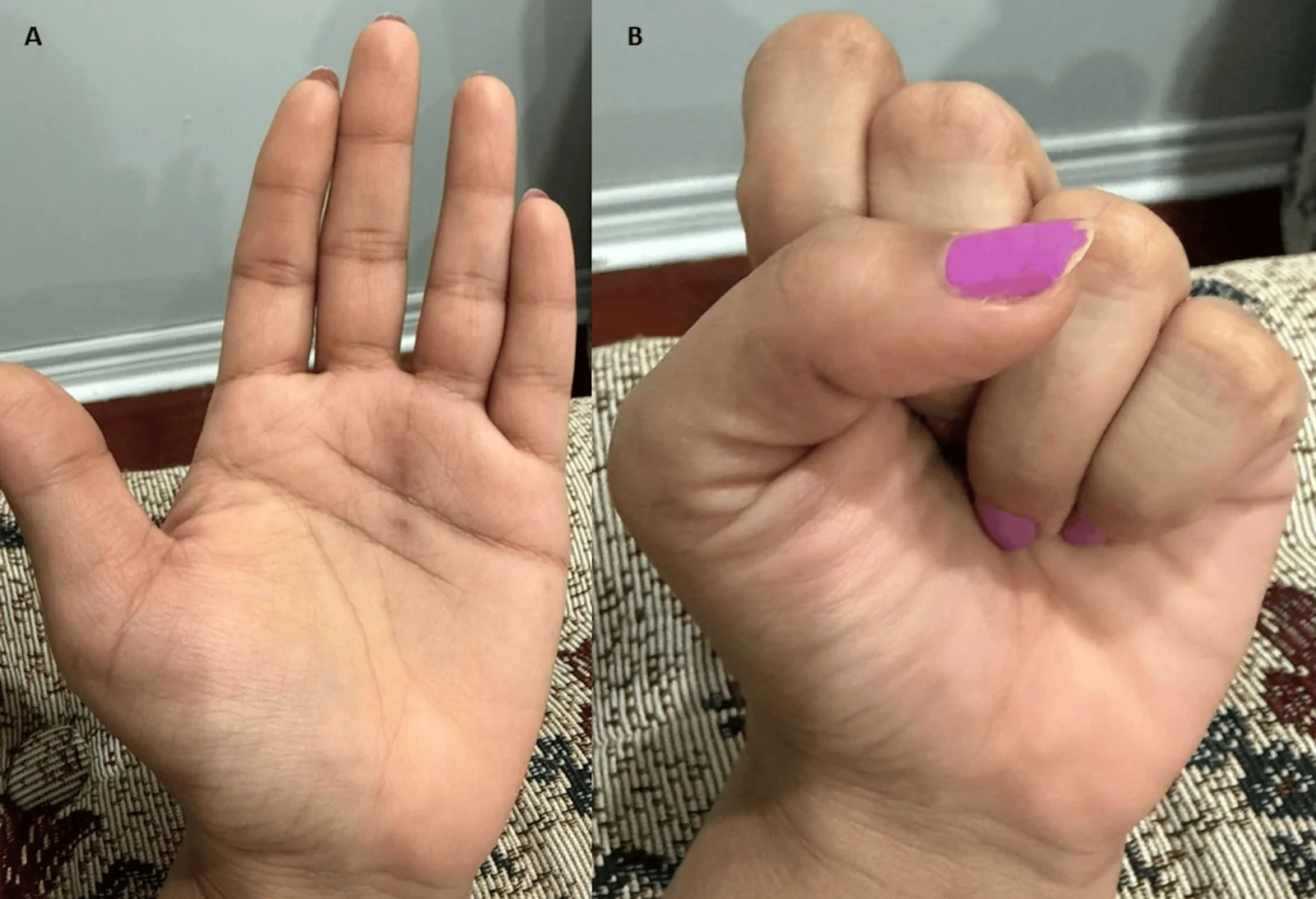
Postoperative outcome (palmar swelling at the base of the ring finger) (A) No evidence of recurrence at follow‑up. (B) Full restoration of hand function with no neurological deficit

Postoperatively, the patient developed transient numbness along the radial aspect of the ring finger, which gradually resolved over three months. Nerve function was assessed by two-point discrimination, light touch, and Medical Research Council motor grading. At one-year follow-up, she had full hand function, with no evidence of recurrence (Figures 5A, 5B).

Case 3

**Figure 6:**
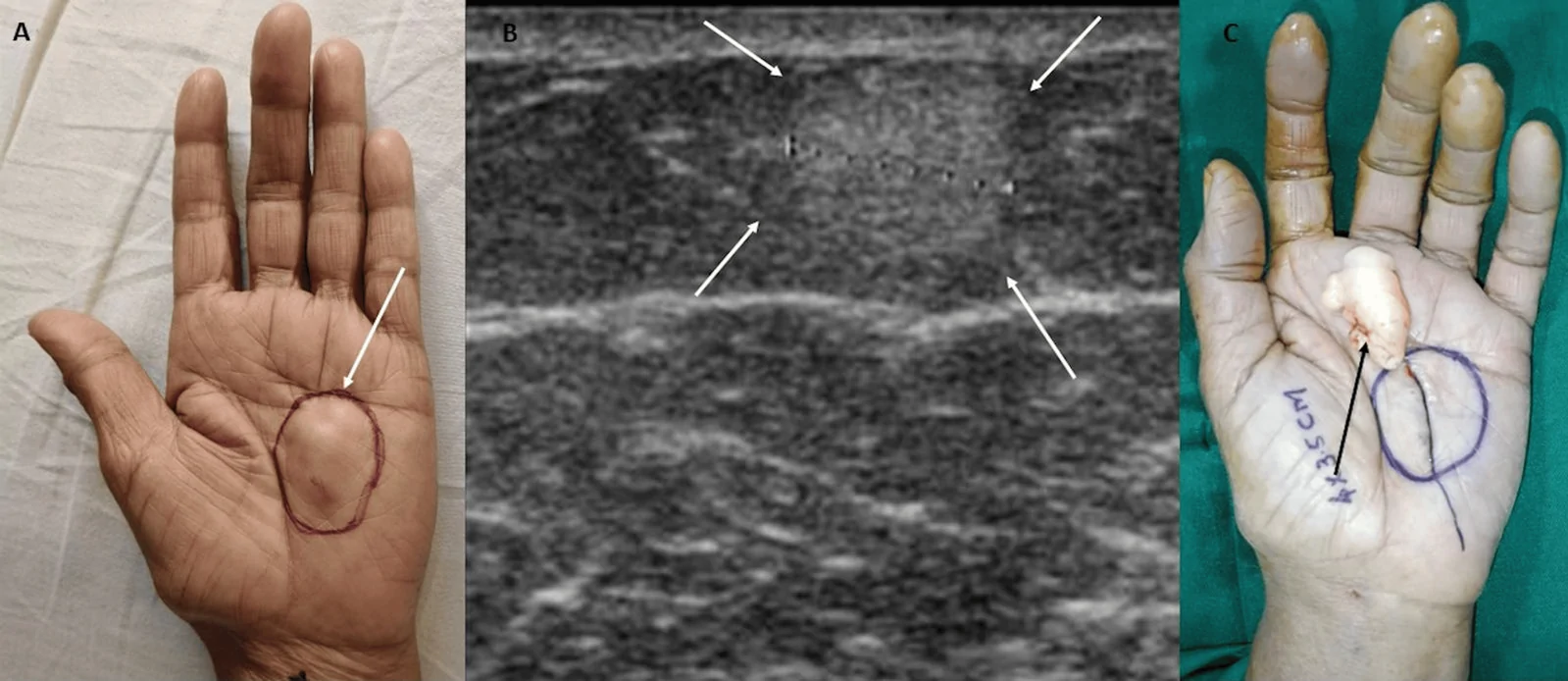
Clinical, radiological, and intraoperative findings of mid palmar swelling (A) Lobulated swelling in the mid-palmar region observed clinically. (B) The ultrasound image shows a well-defined, hypo- to isoechoic, heterogeneous swelling in the subcutaneous tissue. (C) Intraoperative excision demonstrating the gross appearance of the tumor, and the arrow shows the origin from a nerve fiber

A 68-year-old female patient presented with a two-year history of a painful, gradually enlarging swelling in the mid-palm of the left hand (Figure 6A). She was hypertensive, which was well controlled. She had no other comorbidities. The swelling measured approximately 4 × 3.5 cm. The mass was firm and well-circumscribed. She complained of discomfort during gripping. Clinical examination revealed intact distal neurovascular function. Cutaneous stigmata of neurofibromatosis were also observed.

High-resolution ultrasonography demonstrated a well-defined lesion measuring approximately 38 × 20 mm with a predominantly hypo- to isoechoic, heterogeneous internal echotexture (rather than uniformly echogenic), minimal internal vascularity on color Doppler, noncompressibility, no significant posterior acoustic enhancement or shadowing, and apparent continuity with an adjacent nerve suggestive of a lipomatous tumor (Figure 6B). Microsurgical dissection under magnification enabled complete excision while preserving nerve continuity (Figure 6C). Intraoperatively, the lesion arose from the palmar branch of the median nerve, seen entering and exiting a fusiform mass, confirming its neural origin.

**Figure 7:**
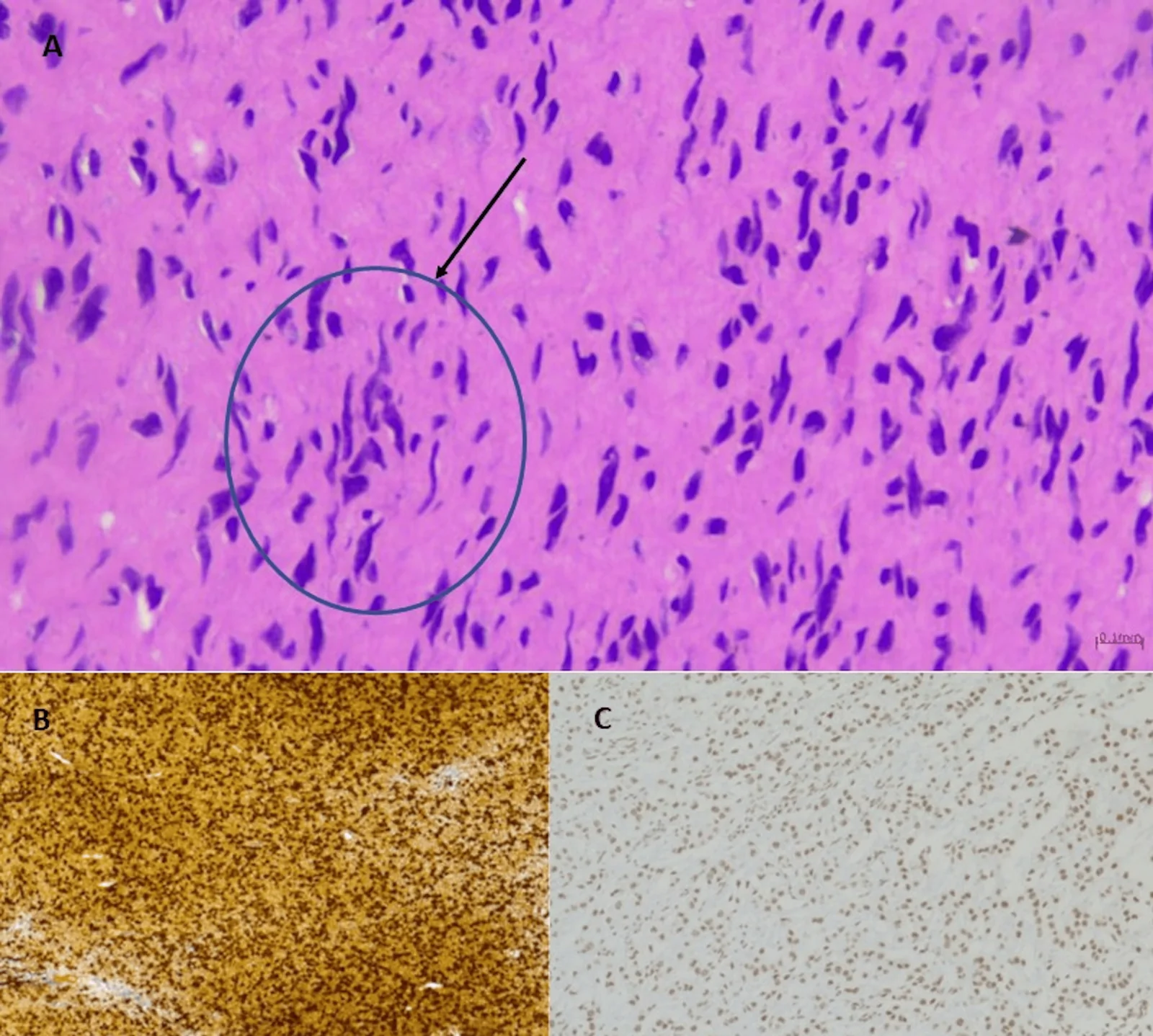
Histopathological picture and images of IHC staining (A) The circle with an arrow shows abundant collagen and wavy, spindle-shaped nuclei after staining the excised tissue from the palm with hematoxylin and eosin stain and viewing with 10× magnification. (B) Numerous neurofibroma cells with S100 IHC stain. (C) Nuclear positivity with SOX10 IHC stain IHC: immunohistochemistry

Histopathological examination revealed a spindle cell lesion composed of elongated cells with wavy nuclei in a collagenous stroma (Figure 7A). IHC demonstrated nuclear SOX10 and S100 positivity in tumor cells, EMA (epithelial membrane antigen) positivity in perineurial cells, and CD34 expression in stromal cells with a lattice network pattern, confirming the diagnosis of neurofibroma (Figures 7B, 7C). The tests were performed using the Dako Autostainer Link 48 (version 4.2.5; Agilent Technologies, Santa Clara, CA): SOX10 (EP268 clone, schwannoma control) showed nuclear positivity in bundled tumor cells; S100 (Beta EP32 clone, schwannoma control) exhibited both cytoplasmic and nuclear staining; and EMA (E29 clone, tonsil epithelial control) highlighted perineurial differentiation.

**Figure 8:**
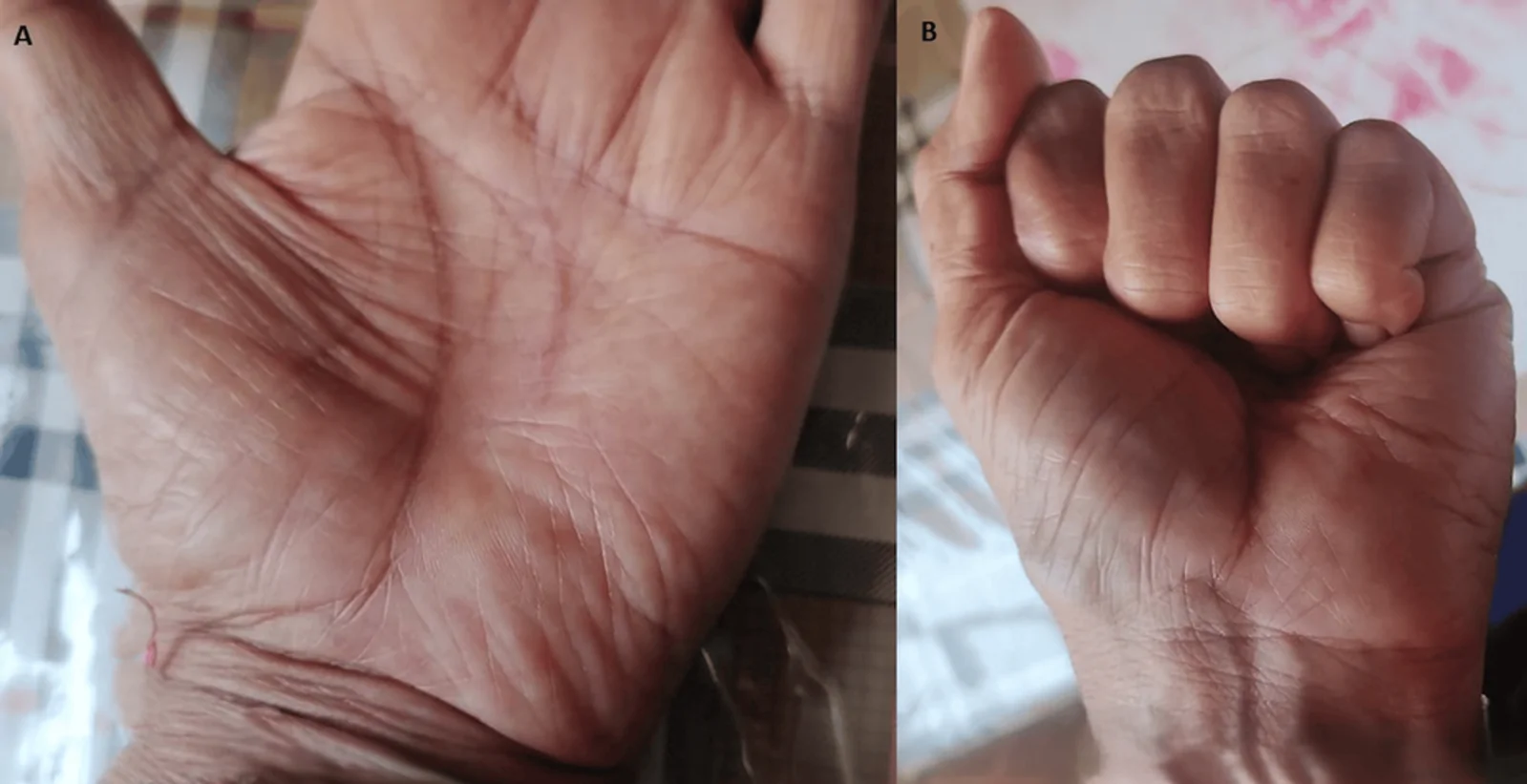
Postoperative outcome (mid-palmar swelling) (A) No evidence of recurrence at follow-up. (B) Full restoration of hand function with no neurological deficit

The postoperative course was uneventful. No motor or sensory deficits were observed. At the one-year follow-up, the patient remained recurrence-free with preserved hand function (Figures 8A, 8B).

The demographic characteristics, imaging findings, immunohistochemical profiles, and clinical outcomes of the three patients are summarized in Table 1. The immunohistochemical panels differed between the cases because staining was performed at different times and laboratories over an eight-year period, with additional markers (SMA in Case 2; EMA in Case 3) added selectively by the reporting pathologist based on the differential diagnosis at the time. Reported staining distribution (patchy vs. diffuse S-100; nuclear SOX10) and appropriate internal/external controls are noted per case.

**Table 1:** Outcomes of patients with palmar neurofibromas initially interpreted as lipomatous lesions

Case	Age/sex	Location	Preoperative imaging diagnosis	Nerve involved	Immunohistochemical findings	Follow-up in years	Outcome
1	30/Male	Thenar palm	Lipoma	Median nerve branch	S-100+, SOX10+, CD34 not performed	8	No recurrence or neurological deficit
2	28/Female	Distal palm, base of ring finger	Lipoma	Digital branch	S-100+, SOX10+, CD34 not performed	1	Transient sensory deficit resolved within 3 months
3	68/Female	Mid-palm	Lipoma	Median nerve - palmar branch	S-100+, SOX10+, CD34+	1	No recurrence or neurological deficit

All lesions were initially interpreted radiologically as lipomatous tumors, whereas definitive diagnosis was established only after histopathological and immunohistochemical evaluation. Complete tumor excision with preservation of neural continuity was achieved in all cases.

All lesions were initially interpreted radiologically as lipomatous tumors; however, definitive diagnosis was established only after histopathological and immunohistochemical evaluation. Complete tumor excision with preservation of neural continuity was achieved in all cases.

## Discussion

Soft-tissue masses of the palm present a diagnostic challenge because of the compact anatomy, diverse histological origins, and overlapping clinical and imaging features of benign lesions. Our series demonstrates that palmar neurofibromas, although rare, can closely mimic lipomas radiologically, underscoring the importance of clinical suspicion and confirmatory histopathology with IHC.

Isolated palmar neurofibromas are exceedingly uncommon, with most reports limited to single cases. Recent reviews also note that these lesions may mimic Dupuytren’s contracture [6]. Their rarity contributes to diagnostic oversight, particularly when imaging suggests a lipomatous lesion. Given the functional importance of the palm, accurate identification is essential to avoid inadvertent nerve injury and facilitate nerve-preserving surgery.

Ultrasonography is commonly used as the initial imaging modality for palmar soft-tissue lesions [9,10]. Lipomas typically appear as well-defined, homogeneous, hyperechoic masses with minimal vascularity, whereas neurofibromas are more often hypoechoic, heterogeneous, and occasionally fusiform along the course of peripheral nerves. However, reliable sonographic differentiation in the palm is limited by its compact anatomy, small lesion size, adjacent tendons and neurovascular structures, and restricted acoustic windows. Consequently, neurofibromas may be misinterpreted as lipomas, especially when classical neural imaging features are absent.

MRI provides superior soft-tissue characterization, although substantial radiologic overlap persists between lipomas and PNSTs [11-13]. Lipomas usually demonstrate homogeneous T1 hyperintensity, uniform fat suppression, and minimal enhancement, whereas neurofibromas tend to show heterogeneous signal intensity with variable enhancement. Findings such as the “target sign,” incomplete fat suppression, fascicular architecture, or continuity with an entering or exiting nerve may suggest a neurogenic tumor; however, these features are inconsistently present. In the confined palmar compartment, identifying the precise nerve of origin is often difficult, creating important surgical implications because lesions presumed to be routine lipomas may instead require meticulous microsurgical nerve-sparing dissection. Supporting these limitations, a prospective study of extremity soft-tissue tumors demonstrated imperfect concordance between radiologic and histopathological diagnoses, emphasizing that imaging alone may be insufficient for definitive diagnosis [14].

The characteristic immunohistochemical differences among lipoma, neurofibroma, schwannoma, and malignant peripheral nerve sheath tumor (MPNST) are summarized in Table 2. These data were derived from previously published studies [7,15,16].

**Table 2:** Characteristic immunohistochemical profiles of lipoma, neurofibroma, schwannoma, and MPNST MPNST: malignant peripheral nerve sheath tumor

Marker	Lipoma	Neurofibroma	Schwannoma	MPNST
S-100	Negative or very weak	Positive (patchy to diffuse)	Strong, diffuse positive	Variable, often focal, or weak
SOX10	Negative	Positive	Strongly positive	Variable, often reduced, or focal
CD34	May be positive in stromal cells	Positive in fibroblastic areas	Variable/usually negative	Usually negative
Ki-67	<1% approximately - very low	<3% approximately – low	<5% approximately - low	>10%-15% approximately – high
p53	Negative	Negative	Negative	Often positive (mutated type)

Histopathologically, neurofibromas are characterized by spindle-shaped cells with elongated wavy nuclei embedded in a collagenous and variably myxoid stroma, whereas lipomas consist of mature adipocytes arranged in lobules. IHC further aids classification through lineage-specific marker expression. Neurofibromas typically demonstrate patchy S-100 and SOX10 positivity with associated CD34 co-expression [17]. In contrast, schwannomas usually exhibit strong diffuse S-100 and SOX10 staining and are generally CD34 negative [7,18]. Recent studies also suggest that CD56 and calretinin may assist differentiation, as these markers are commonly positive in schwannomas but absent or minimally expressed in neurofibromas [19].

Distinguishing neurofibroma from other benign and MPNSTs is clinically important because their management and prognosis differ substantially. MPNSTs typically demonstrate focal or weak S-100/SOX10 expression, loss of CD34 staining, and elevated Ki-67 and p53 indices, reflecting increased proliferative activity and malignant transformation. Ki-67 values are approximate, literature-derived ranges, and overlap between benign and MPNSTs. Ki-67 value alone cannot reliably distinguish neurofibroma from MPNST. One must remember that immunohistochemical markers are supportive and should be interpreted in conjunction with morphology, rather than being independently diagnostic. The correlation of morphologic and immunohistochemical findings, therefore, remains essential for accurate diagnosis, exclusion of malignant mimics, and appropriate postoperative management.

Differentiating neurofibromas from lipomas is also surgically important because the operative strategy and functional outcome depend on recognizing neural involvement. Failure to identify a PNST preoperatively or intraoperatively may result in traction injury, fascicular damage, incomplete excision, or postoperative neurological deficits. Clinical features such as pain, firmness, adherence to deeper tissues, proximity to neurovascular structures, or atypical imaging characteristics should therefore raise suspicion for a neurogenic lesion and prompt magnification-assisted microsurgical dissection rather than routine excision [20].

In our series, all lesions were initially interpreted radiologically as lipomas, highlighting the limitations of imaging alone in small palmar soft-tissue tumors. Definitive diagnosis relied on postoperative histopathological and immunohistochemical evaluation, which also enabled reliable distinction from schwannomas, lipomas, and MPNSTs. Nevertheless, preoperative awareness of this possibility was valuable in guiding careful nerve-sparing surgical techniques and avoiding iatrogenic injury.

Appropriate preoperative counseling remains an important component of management. Patients should be informed about the possibility of neural involvement, the risks of temporary or permanent sensory deficits, and the potential need for nerve-related procedures depending on intraoperative findings. Such counseling facilitates informed consent and helps align postoperative expectations.

When a palmar soft-tissue lesion demonstrates pain, neural symptoms, fusiform morphology, incomplete fat suppression, or continuity with a nerve on MRI, a PNST should be strongly considered, despite an apparently lipomatous appearance.

**Figure 9:**
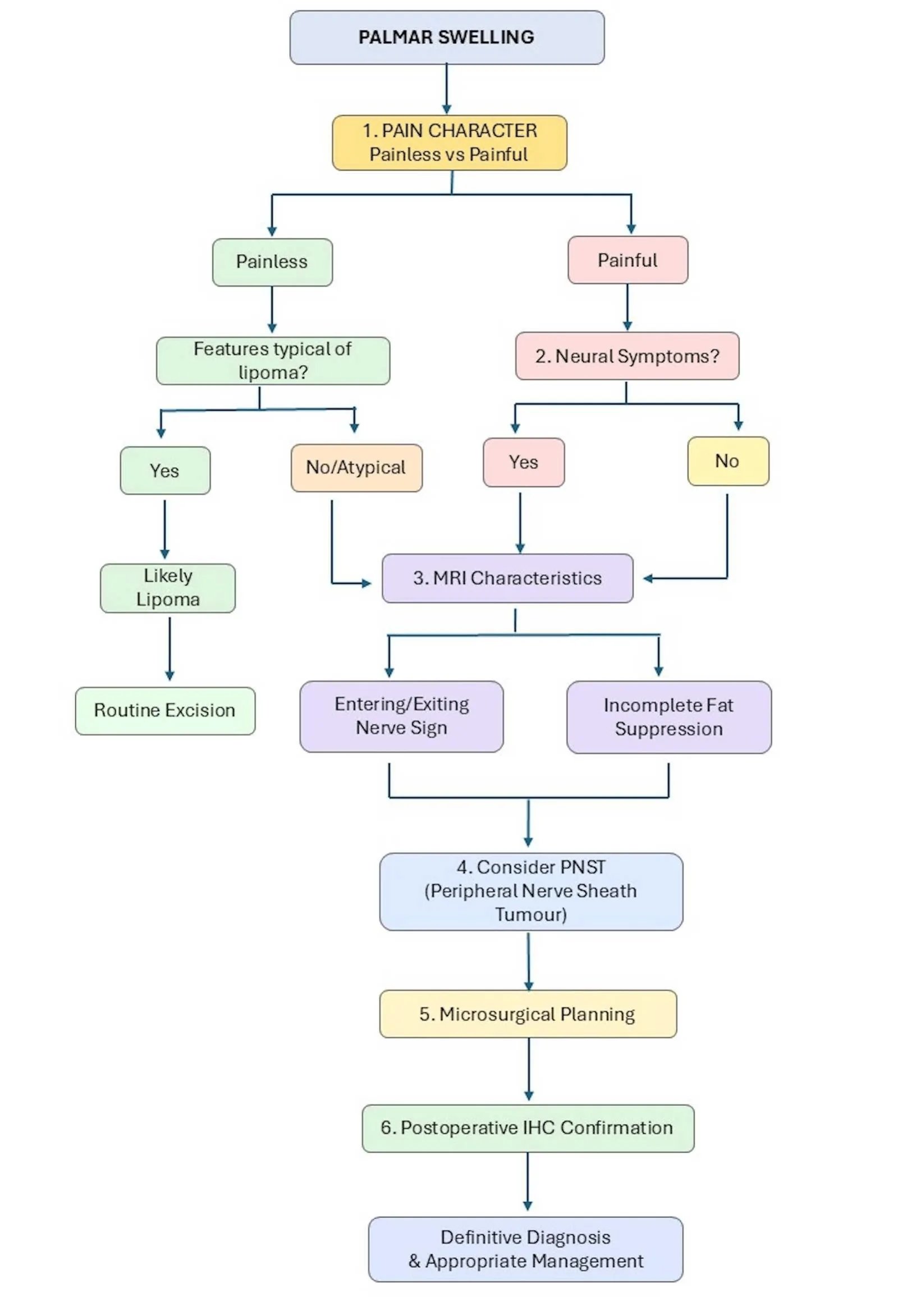
Diagnostic and management algorithm for painful palmar swellings with possible PNST features Algorithm illustrating the evaluation of palmar soft-tissue swellings and the identification of features suggestive of a PNST. Clinical findings (pain and neural symptoms) and MRI characteristics (entering/exiting nerve signs or incomplete fat suppression) guide the suspicion for a neurogenic lesion and the need for magnification-assisted nerve-sparing excision. The definitive diagnosis was subsequently confirmed by histopathological examination and IHC MRI: magnetic resonance imaging; PNST: peripheral nerve sheath tumor; IHC: immunohistochemistry Image credit: This figure was generated using Microsoft PowerPoint 2016 (Microsoft Corporation, Redmond, WA)

We propose a diagnostic and management algorithm as a preliminary, nonvalidated, hypothesis-generating tool based on limited evidence, which requires prospective validation (Figure 9). The algorithm is designed to facilitate clinical decision-making, outlining recommended investigations and management strategies when a neurogenic lesion is suspected. Because even painless lesions with apparently typical lipomatous imaging may occasionally be neurogenic, the “routine excision” pathway is qualified to advise intraoperative vigilance for a nerve of origin in all cases.

Limitations

This study has several limitations. The sample size is small owing to the rarity of isolated palmar neurofibromas, limiting generalizability, and the retrospective single-center design may introduce selection bias. Imaging protocols and radiologic interpretation were not fully standardized. The “lipoma” impression was taken from the original prospective reports of fellowship-trained musculoskeletal radiologists rather than assigned retrospectively, and a centralized blinded imaging re-review was not performed. Objective functional instruments (grip dynamometry, Disabilities of the Arm, Shoulder and Hand/Quick Disabilities of the Arm, Shoulder, and Hand) were not consistently recorded. Diagnoses were based on the original pathology reports and available archived material. For Case 1, the slides and block were discarded per routine policy, and a contemporary blinded histological/immunohistochemical reassessment could not be performed. Genetic evaluation for neurofibromatosis was not performed. Larger multicenter studies with standardized protocols, objective functional assessment, and longer follow-up are required to validate these observations.

## Conclusions

Palmar neurofibromas are rare lesions that can closely resemble lipomas and other benign soft-tissue masses of the hand, creating significant diagnostic uncertainty. In this series, all three lesions were initially interpreted as lipomatous tumors on clinical and radiological grounds. The diagnosis of neurofibroma was confirmed only after histopathological examination supplemented by IHC. These observations highlight the need to maintain a broad differential diagnosis when evaluating atypical palmar masses.

Complete excision with nerve preservation was achieved in all three patients, with no recurrence observed during the available follow-up. Histopathology and IHC are indispensable for definitive diagnosis and for distinguishing neurofibroma from schwannoma, MPNST, and other soft-tissue neoplasms. Greater recognition of this uncommon presentation may improve preoperative planning, patient counseling, and the overall management of peripheral nerve tumors of the hand.
